# A Guide to Designing a Memory fMRI Paradigm for Pre-surgical Evaluation in Temporal Lobe Epilepsy

**DOI:** 10.3389/fneur.2019.01354

**Published:** 2020-01-09

**Authors:** Sarah Buck, Meneka K. Sidhu

**Affiliations:** ^1^Department of Clinical and Experimental Epilepsy, UCL Institute of Neurology of Neurology, London, United Kingdom; ^2^Epilepsy Society MRI Unit, Chalfont Saint Peter, United Kingdom

**Keywords:** fMRI, memory, TLE, paradigm, guide, method, recall, recognition

## Abstract

There has been increasing interest in the clinical and experimental use of memory functional Magnetic Resonance Imaging (fMRI). The 2017 American Academy of Neurology practice guidelines on the use of pre-surgical cognitive fMRI suggests that verbal memory fMRI could be used to lateralize memory functions in people with Temporal Lobe Epilepsy (TLE) and should be used to predict post-operative verbal memory outcome. There are however technical and methodological considerations, to optimize both the sensitivity and specificity of this imaging modality. Below we discuss these constraints and suggest recommendations to consider when designing a memory fMRI paradigm.

## Introduction

The most important cognitive comorbidity of TLE is impairment in episodic memory. The hippocampus plays a major role in the generation and spread of temporal lobe seizures (1), and it is also a critical structure serving long-term memory, including episodic memory (2). It therefore follows that impairments in memory and learning are frequently seen in people with TLE, although more wide-spread cognitive deficits have also been reported (3). In up to 80% of TLE, epilepsy surgery can be curative (4), however, cognitive decline remains a significant complication of epilepsy surgery (5–10). Early onset seizures interfere with the normal process of hemispheric lateralization (11) and may result in the reorganization of memory functions (12–14). In unilateral TLE, both the function of the contra-lateral MTL (hippocampal adequacy theory), and functional reserve of the ipsilateral hippocampus have been posited in the maintenance of post-operative memory functions (15). It is therefore important to identify the lateralization and localization of memory functions prior to surgical intervention to evaluate the risk of significant post-operative memory deficits.

Memory functional magnetic resonance imaging (fMRI) has been used to study the localization and functional lateralization of critical structures involved in the specific memory task employed (16–22). Memory fMRI is also useful in the prediction of post-operative memory performance (20, 23, 24). Encouragingly, memory fMRI was shown to be the strongest independent predictor of post-operative memory decline compared to standard clinical outcome predictors such as age at onset of epilepsy, hippocampal volume, and pre-operative neuropsychometry (14, 20). Memory fMRI has also been used to investigate post-operative memory plasticity (25–28).

However, memory fMRI remains challenging due to several neuropsychological and technical considerations. Heterogeneous findings across memory fMRI studies may relate to methodological differences, particularly with regards to the memory task itself. This is reflected in the failure to replicate results and paucity of MTL activations in some studies. The quality of fMRI data depends on several factors including paradigm design, task selection, data acquisition and analysis (29).

We aim to provide a guide for clinicians and researchers to design a memory fMRI protocol for pre-surgical evaluation of memory in TLE. This guide will help the readers with paradigm selection (section Paradigm selection), data analysis (section Analyses), and improvement of reliability of fMRI data (section Reliability of fMRI Data). The section on Paradigm Selection describes the different cognitive processes involved in memory. Understanding these processes will help the readers identify which process they wish to specifically study. Examples of paradigms are mentioned for each of those memory processes. Section Analyses discusses inter-subject variance in brain activation and its implication for interpretation of individual-subject data. In this section, event-related and block analyses are also discussed, as they should guide the design of the paradigm. Finally, the section on reliability of fMRI data discusses issues related to poor reliability of fMRI data and suggests ways to improve it. We hope that through this guide, the reader gains awareness in the parameters to consider when designing a memory fMRI paradigm. This guide is primarily for adults. Whilst similar principals apply in pediatrics, paradigm length and complexity may need to be adjusted according to the age of the participant.

## Paradigm Selection

### Neuropsychology

Brain activation can vary depending on the nature of the memory task and other cognitive demands related to the task. For this reason, good understanding of the cognitive processes involved in memory is important when designing an fMRI protocol.

#### Associative Memory and the Hippocampus

Surgical intervention involves the resection of the temporal lobe lesion and the epileptogenic zone which usually encroaches on the hippocampus (30, 31). Pre-surgical investigation of a patient's functional anatomy surrounding the brain lesion is therefore critical for the surgical approach, and designing a memory fMRI task for which performance is supported by the hippocampus appears most relevant in this case.

It is well-recognized that the hippocampus is critical for the binding of information into a representation for later retrieval, as required in paired-associate learning tasks (7, 32–36). The hippocampus contributes to associative memory, whereas other non-hippocampal medial temporal regions contribute to single-item memory (32, 33, 37). Patient studies have demonstrated that the effects of lesion to the hippocampus are selective to specific forms of memory, and are apparent on tasks of arbitrary paired-associates (38), in which association between the items of a pair is necessary for successful performance. Given the role of the hippocampus in TLE, a paired-associate memory paradigm, such as word pairs (39) or face-name associations (40–42), may be most appropriate for the investigation of hippocampal-dependent memory.

#### Memory Formation

Memory formation is a complex dynamic process that is carried out by representational systems in the brain; distinguished by the nature of the information and task presented (43). Long-term memory is made up of explicit (declarative) and implicit (non-declarative) memory systems (44). Explicit memory allows conscious recall and is sub-divided into semantic memory; the conscious recall of factual knowledge, and episodic memory; recall of individual events in spatial and context order. Critical steps of episodic memory include the formation of distinct neural traces during memory encoding, memory storage and memory retrieval (45).

The first stage of memory is encoding, whereby the information is perceived and transformed into a mental representation. Retrieval is the process by which information that is stored in memory is re-accessed. Retrieving information from memory can occur through the processes of recollection and familiarity. Recollection refers to the reliving of vivid and detailed episodes, whereas familiarity is associated with a sense that information was previously encountered but without any contextual detail. These two processes are mediated by distinct sub-regions of the MTL. Recollection is supported by the hippocampus, and familiarity, the perirhinal cortex (46–48).

The frequently used “Old/New” paradigm compares brain activation associated with the retrieval of studied items (“Old”) and new items (“New”). A potential drawback with these paradigms relates to the fact that brain activation associated with the retrieval of studied items could reflect either familiarity or recollection processes. This could lead to inaccurate conclusions regarding the differential role of sub-regions engaged within the MTL.

Paradigms that involve recollection processes are more likely to engage the hippocampus. This can be achieved using the “Remember/Know” paradigm (49–51) for which the responses are thought to reflect recollection and familiarity processes, respectively (52). However, familiarity and recollection may differ along a continuum depending on response confidence, and the imaging contrasts may not accurately reflect the underlying cognitive process (48). Brain activation during a so-called “recollection” contrast (i.e., “Remember>Know”) may also include some activity related to familiarity; leading to variability in fMRI studies. In imaging studies, paradigms like the “Old/New” or “Remember/Know” can be adapted to either measure brain activity during the encoding phase or the retrieval phase of the memory process. These are described below.

##### Memory encoding

Memory fMRI studies often evaluate the encoding phase, with retrieval assessed after scanning (14, 17, 20, 22, 24, 53). Images are acquired during the presentation of information, when participants are encouraged to memorize items presented in the scanner, with retrieval of information occurring after the scanning session.

For example, in the “Old/New” paradigm first described by Powel et al. (54), used by Bonelli et al. (20), and adapted by Sidhu et al. (14, 22, 24), verbal and visual items are visually presented to the subjects during the scanning session. Subjects perform a deep encoding task which involves making a judgement on whether each presented stimuli is pleasant or unpleasant. After the scanning session, subjects perform a recognition test outside the scanner. During this test, the previously presented stimuli are randomly mixed with foils. For each item, subjects are instructed to indicate whether they remember seeing each stimulus during scanning, or whether it is new. The stimuli presented during scanning are then classified according to the responses made during the recognition test. A correct response indicates that the stimulus was subsequently remembered, whereas an incorrect response indicates that the stimulus was subsequently forgotten. Sidhu et al. later included a third response option (“Familiar”) to distinguish between the processes of recollection (“Remember” response) and familiarity (“Familiar” response). This type of paradigm provides information about the neural network associated with the encoding phase of memory, but not the network that is involved in the retrieval of mnemonic information.

##### Memory retrieval

Paradigms that map the retrieval-related network can involve recognition- or recall-based retrieval, as described below.

##### Recognition

Recognition reflects the ability to identify presented items as familiar, and as such rely on familiarity processes and can be performed without involvement of the hippocampus. FMRI studies investigating retrieval-related activations often use a recognition task (for example “Old/New” or “Remember/Know” paradigms, as described above) (55), and examine MTL activity during successful recognition.

Smith et al. (51) used a “Remember/Know” paradigm which involved studying and making pleasant/unpleasant judgments to words prior to the scanning session. Twenty minutes after studying the words, subjects took a memory test inside the scanner which included the studied words along with foils. For each words presented inside the MRI scanner, subjects made an old/new judgment using a 20-point scale (1 = definitely new, 20 = definitely old). For words identified as “old,” subjects were further asked to indicate whether the word was recollected, familiar, or a guess. Participants were instructed to use the “remember” response only if they could describe specific details about the experience of studying the word and to use the “familiar” response if the word was familiar but they could not retrieve contextual details. Subjects provided their responses inside the scanner by moving an MRI-compatible mouse to the relevant location on the screen. Whereas, recognition paradigms like the one described above are often used in memory fMRI studies, they lack in the ability to identify recall-related processes.

##### Recall

Recall refers to the ability to bring back to mind consolidated representations and relies on recollection processes. Based on evidence from patients with bilateral hippocampal damage of early onset, it is recognized that the hippocampus supports recall processes (56). Recall-based memory should be considered in memory fMRI studies to represent ecological scenarios of every-day memory process, and to optimize hippocampal activation.

During recall, a fragment of the pattern representing the event from the neocortical system triggers the retrieval of the whole representation via pattern completion supported by the hippocampal system (57). In order to recall an event (to bring back to mind a specific past episode), the different features of the event must be processed and bound together. Successful recall therefore requires the use of associative mechanisms, which depend on the hippocampus (58, 59).

Reas et al. (39) used a recall-based memory fMRI paradigm whereby subjects studied word pairs prior to the scanning session. After study, subjects were given a self-paced cued recall test where they were presented with one word of each pair and were asked to say out loud the word that was paired with it. Forgotten pairs were repeated until all pairs were successfully recalled. After a 20-min delay, subjects performed a recall and classify task inside the MRI scanner. They were presented with one word of each pair and were instructed to covertly recall the missing word and to classify it as living or non-living. A third response option (“unsure”) was given if they did not remember the pair of the presented word. Subjects provided their responses inside the scanner using a four-button response box. Following the scan, subjects performed a self-paced cued recall test to evaluate the retrieval success of the in-scanner cued recall task.

To date, fMRI studies that use such recall paradigms usually involve covert responses, with additional verbal recall after the scanning session to measure performance (39, 60). A potential issue with this approach is that performance may differ between the two retrieval periods, and the fMRI data may therefore not fully represent activation related to successful performance. In this respect, in-scanner overt recall may be more valid (see section Overt Responses).

##### Combined encoding and retrieval paradigm

The specific memory process that is impaired in the patient group should guide the selection of the paradigm. People with TLE may have difficulty with both encoding and retrieval of information. As such, studying the mechanisms of both encoding and retrieval (41, 61) may be useful. An fMRI protocol that maps both the encoding and retrieval phases of the memory process could provide a more robust mapping of memory-related networks, as both phases are dependent on hippocampal involvement (62, 63). Obtaining robust hippocampal activation at the individual level has proven challenging across fMRI studies (63, 64), but a wider approach to memory mapping involving two memory phases (encoding and retrieval) may increase the sensitivity of this.

### Aim of the Protocol

The clinical aim of the study is pertinent in paradigm selection. If the aim is to study re-organization of memory functions to the contralateral MTL, a material-specific paradigm would need to be employed. Verbal material activates the dominant hemisphere and visual, the non-dominant hemisphere (11, 65, 66), whereas bilateral tasks such as picture or scene encoding incur bilateral MTL activations. “Failure of activation” using these bilateral tasks have been used to test the hippocampal adequacy vs. the functional reserve model in the prediction of post-operative memory outcome (17).

Irrespective of material type, test-retest reliability of a paradigm is an important consideration (see section Reliability of fMRI Data). In memory-fMRI, reproducible hippocampal magnitude was shown using “hometown walking,” a paradigm which requires imagining a familiar route in the scanner (67). In the same study, test-retest of verbal memory recall most reliably identified hemispheric lateralization, which is clinically pertinent to guide surgical planning and predict memory outcome. The clinical aim of the study should therefore inform the design of the fMRI paradigm.

### Overt Responses

Most fMRI studies involve covert verbal responses in order to avoid speech-related artifacts (21, 68). However, overt verbal responses may be advantageous for clinical studies, as they are useful to monitor in-scanner performance and conduct event-related analyses (see section Event vs. Block Analysis). Involving overt responses allows online measure of performance and this is beneficial as it makes it possible to explore specific brain activation associated with verbal output. This is particularly relevant for the interpretation of performance and the investigation of brain networks in people with cognitive impairment. The associated movement-related artifacts can be controlled for using image processing techniques (69). Studies have employed overt cued-recall paradigms and demonstrated significant activation in the MTL for successful recall (61, 70, 71). Overt responses should therefore be considered in memory fMRI paradigms.

### Baseline Task

Baseline tasks are subtracted from the active memory conditions to generate “activation contrasts.” In memory studies, there are two main considerations in selecting a baseline task. The first, is to model a pure memory process. For this, an active baseline task that removes attention, language and motor processes should be considered (72). Next, the baseline task should not activate the hippocampus as this would reduce the sensitivity of hippocampal activations associated with the active process, when contrasted. In a study comparing several baseline tasks, Stark and Squire (73) demonstrated higher activation in the hippocampal region associated with a memory task when the odd/even digit task was used as baseline, compared to when rest was used as baseline.

**Figure 1:**
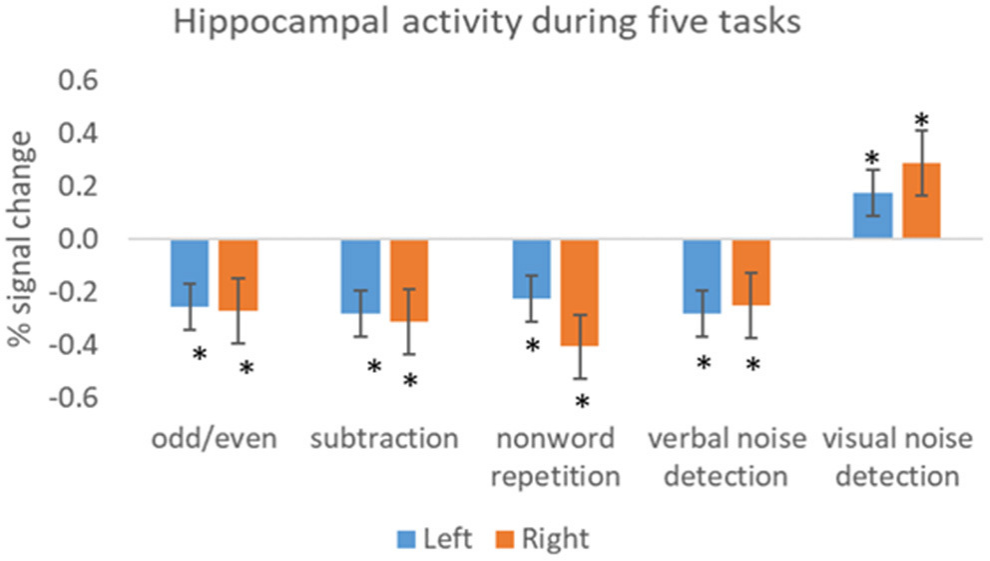
Activity within the hippocampus (left and right) during five baseline tasks. The bars show the mean percent signal change during each task relative to the mean signal during rest. The results show significant less activation in all tasks, except visual noise detection, compared to rest. Error bars = SEM. Significant at *p < 0.05.

As in active tasks, baseline task activations vary. We examined hippocampal activation in three healthy participants during five baseline tasks, compared to rest. These included: an odd/even number task where participants were presented with double digits and were asked to decide whether the number was odd or even; an arithmetic subtraction task (for example 27–4); a non-word repetition task where participants were visually presented with two syllable non-words and were asked to read them out loud; a verbal noise detection task where participants were asked to indicate whether mixed letters were presented in pale green or blue and a visual noise detection task where participants were asked to indicate whether shapes, which were embedded in a visual white noise mask, were presented in pale green or blue. All the stimuli were presented every 2 s, apart for the subtraction task where stimuli were presented every 3 s. Compared to rest, there were significantly less bilateral hippocampal activations in all baseline tasks except for visual noise detection (Figure 1). These four baseline tasks are therefore ideal for maximizing hippocampal activations when subtracted from an active memory task. Careful piloting of both the active and baseline tasks is therefore recommended when designing a memory fMRI paradigm.

## Analyses

### Single-Subject Level vs. Group-Level Analyses

Group analysis collapses data across subjects and examines the overlapping effects. Variability between subjects is considered as nuisance and is included as covariate in the model to make group inferences. One example of inter-subject variability is differences in cognitive strategy for the same task. Each strategy is associated with specific cognitive processes and leads to distinct activation pattern. However, group analyses assume that the task is performed using the same strategy across individuals, and inter-subject variability is ignored. Seghier and Price (74) argued that such between-subject variance should be treated as data rather than noise, particularly in the field of psychology [see (Seghier and Price) for a guide on how to model inter-subject variance]. Understanding normal inter-subject variance can help understand differences in cognitive outcomes between patients and optimize the full potential of neuroimaging applications. Moreover, inter-subject variance may be related to behavioral functions (75, 76) and could provide useful clinical information with regards to predicting outcome in a patient population. Whereas, group studies examine mean effects across subjects, inter-subject variance may provide critical information, and should not be ignored.

With high variability in the pattern of brain activation, the question arises as to how a subject's memory activation can be interpreted in single-level analysis. Multivariate Pattern Analysis (MVPA) can be carried out on fMRI data to examine the distributed pattern of activation across voxels at the individual-subject level (77). MVPA exploits voxel-level variability within subjects and neutralizes the effects of subject variability, and is therefore more sensitive to neural differences at the individual level than univariate analyses.

A translational application of memory fMRI is to be able to use this to guide surgical planning in TLE. This is only possible if fMRI activations are valid at the single-subject level, and should be considered when examining validity of a novel fMRI tool. Further research is required to assist single-subject fMRI for clinical purposes.

### Event vs. Block Analysis

Individual fMRI activations also vary depending on the choice of analysis (i.e., block- or event-related analysis). Block analyses allow examination of brain activity related to memory effort, irrespective of performance, whereas event-related analyses specifically examine successful memory formation. The latter is particularly relevant for predicting memory outcome in the clinical setting. In block analyses, memory and baseline conditions are separated into blocks of extended time intervals. Block analyses have a higher sensitivity (78), meaning that it has good ability to differentiate between different conditions. In event-related analyses, the Blood-Oxygenated Level Dependent response is modeled to each trial within a block (79). It allows the separation of trials based on the participant's performance, for example remembered vs. forgotten items. It provides a better representation of the latency of brain response by providing a better characterization of the shape and the onset of the hemodynamic response function than block analyses (80).

The type of analysis (block vs. event) should be considered prior to designing the fMRI paradigm as the design will depend on the analysis of interest. Maus et al. suggested an optimum block length of 15 s for block analysis (81), and a decrease in percent signal change was shown with longer blocks (82). By contrast, block length is less pertinent for event-related analyses. Block lengths should also take into consideration task-related cognitive demands. Too long or too “difficult” tasks could lead to reduced attention and performance, significantly impacting on the quality of data obtained.

Particularly in event-related analysis, the question of task difficulty is critical. For reliable fMRI activations, it is absolutely vital that participants are able to perform a task. In memory fMRI, the contrasts investigate brain activation for remembered vs. forgotten items, and as such enough trials are needed in each condition. Consider a task that is too difficult, most items will be forgotten, with very few “remembered” trials. In this case, the contrast “remembered vs. forgotten” will not accurately identify the successful memory network. The question of task difficulty is pertinent in pediatric and patient studies where ability levels vary considerably. The paradigm should be designed to reach levels of around 50% correct performance to allow inter-subject performance variability whilst avoiding floor/ceiling effect. Patient factors such as degree of cognitive impairment are therefore important considerations. A trade-off between optimal hippocampal activations, length of scan and paradigm complexity should be sought. With these considerations, the choice of block- or event-related analyses should be made prior to paradigm selection, as the design of the paradigm will depend on the analyses.

### Other Analyses

Connectivity techniques investigate functionally connected brain regions involved in a task at a specific time [see (83) for a review]. This allows for the assessment of memory processes at the network level. Multivariate pattern analysis applied to fMRI data [see (84) for a review on the technique] focuses on the patterns of activity (rather than individual activations) across voxels in specific brain regions that are associated with individual memory traces (85–87). A detailed discussion of these techniques is out of the scope of this manuscript (88).

## Reliability of fMRI Data

Test-retest reliability of fMRI findings is rarely investigated, and studies that investigate it generally report poor reliability of brain activations [see (89), for a review]. This significantly impacts on the clinical utility of the paradigm. Despite advances in hardware and fMRI techniques, the sensitivity and therefore reliability of single-subject fMRI remains sub-optimal (90). Brandt and colleagues investigated reliability of memory fMRI activation using data from two sessions, 1 month apart. The authors measured Intra-Class Correlation (ICC) for the degree of activation at each voxel of the brain and reported that despite reliability of memory activation at the group-level, activation was not stable within individuals (91).

ICC is a measure generally used and represents the ratio of between-subject variance and between-tests variance. ICC can be easily computed using statistical analysis software such as SPSS, by running “Reliability Analysis” (under “analyse,” then “scale”) and checking “InterClass correlation coefficient.” The value approaches 1 if the individual variability is low, and an ICC of 0.5 is considered largely concordant in fMRI studies (89).

**Figure 2:**
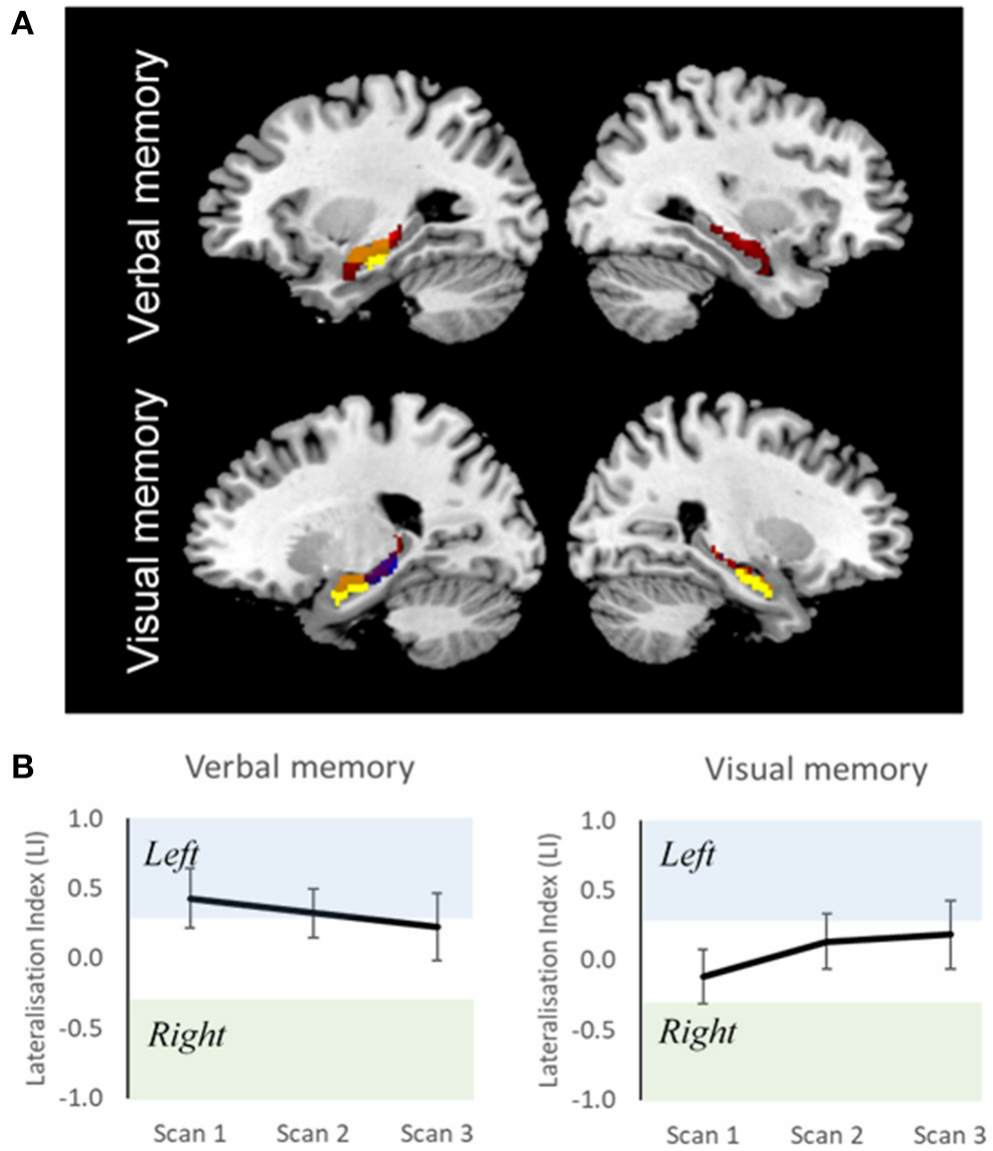
Reliability of fMRI data in healthy individuals. **(A)** Overlap of group activation maps for three fMRI sessions, for verbal and visual memory separately (*p* < 0.001), masked around the hippocampi. Yellow, session 1 (time 0); blue, session 2 (time 0 + 5 months); red, session 3 (session 2 + 10 months); orange, overlap between session. **(B)** Group-Level Lateralisation Indices for verbal and visual memory across three fMRI sessions (95% CIs). Scan 1, time 0; scan 2, time 0 + 5 months; scan 3, scan 2 + 10 months. Despite overlap of brain activation between sessions, variation in functional lateralization is observed. This shows the importance of acquiring control scans at similar time points to patients in longitudinal studies.

Measures of reliability for the magnitude and extent of activation and for the lateralization of activations have been reported in several studies (61, 67, 91–94). Buck et al. measured reliability of memory retrieval lateralization across two separate sessions, 1.5 years apart, and demonstrated good inter-session reliability, suggesting its promising use in single-subject level analysis (61). In our current data, we looked at the ICC of 15 healthy controls scanned across three time-points. Although there was overlap in MTL activations across the three sessions, the spatial extent differed (Figure 2A). ICC for LIs across fMRI sessions were more stable for verbal (0.65) compared to visual (0.35) memory (Figure 2B). This is in keeping with previous reported ICC studies. Given the test-retest variability of fMRI activations, longitudinal studies in people with TLE should be contrasted with those of healthy controls scanned across the same time-points. Performing a mixed ANOVA using a flexible factorial design (97) can be used to model changes in activation at the different time-points whilst controlling for between-subjects and between-group variance in a single model (27).

Regions within the MTL are particularly susceptible to poor reliability of brain activation (91), which has important implications with regards to interpreting fMRI results. Several factors can however improve reliability of fMRI results, including increasing the size of the regions of interests (95), having additional runs (96) and increasing the signal-to-noise ratio by having additional scans (89). Keeping physiologic functions as uniform as possible such as amount of sleep and time of day of scanning are also important considerations.

## Difficulties With Memory fMRI

fMRI involving MTL structures is subject to distortions due to the inhomogeneous magnetic field. MTL susceptibility artifacts lead to image distortion and signal loss (98), making it difficult to obtain reliable signal thereby, hampering interpretation. For these reasons, methodological considerations need to be rigorously applied in fMRI studies that have a particular interest in the MTL. For example, a slice tilt can be applied to align the scans perpendicular to the long axis of the hippocampus and optimize the Blood Oxygenated Level Dependent sensitivity in medial temporal lobe regions (99).

Moreover, fMRI is susceptible to motion artifact as a result of long acquisition time. fMRI detects signal changes in an image over time (i.e., changes in neural activity), but head motion can be misinterpreted as relevant change. It has been shown that patients (100) and children (101) have particular difficulty remaining still inside the scanner, for whom motion artifacts are therefore particularly apparent. fMRI brain mapping is therefore limited by several factors which alter interpretation of fMRI findings. However, careful considerations related to paradigm selection (as described in the present guide), as well as data acquisition and data processing can be implemented to reduce or counteract these limitations [see (88) for a guide on pre-processing and analysis of fMRI data].

## Conclusions

There has been accruing evidence for the clinical utility of memory fMRI in the pre-surgical assessment of people with TLE. The ultimate aim is to acquire reliable and sensitive data not just at the group level but also at the single-subject level for translational clinical application. There is no single “gold standard” memory fMRI protocol due to the variability in parameters to consider, such as specific memory process of interest and cognitive ability of patients. However, considering the involvement of the hippocampus in TLE, we recommend paradigms of associative memory (for the binding of information which is dependent on the hippocampus) or paradigms that involve encoding and recall (rather than recognition). We also discussed the advantages of overt responses, despite motion-related artifacts, for in-scanner monitoring of performance and for the application of event-related analysis. We hope this guide will be of assistance in identifying the specific paradigm and parameters to those who wish to design a memory fMRI paradigm for clinical or research purposes.

## Data Availability Statement

The datasets generated for this study are available on request to the corresponding author.

## Ethics Statement

The studies involving human participants were reviewed and approved by National Hospital for Neurology and Neurosurgery and the Institute of Neurology Joint Research Ethics Committee, and London-Stanmore Research Ethics Committee. The patients/participants provided their written informed consent to participate in this study.

## Author Contributions

All authors listed have made a substantial, direct and intellectual contribution to the work, and approved it for publication.

### Conflict of Interest

The authors declare that the research was conducted in the absence of any commercial or financial relationships that could be construed as a potential conflict of interest. The handling Editor declared a shared affiliation, though no other collaboration, with one of the authors SB.
